# Cardiac allograft vasculopathy in heart transplanted recipients: The multivessel study

**DOI:** 10.1016/j.jhlto.2023.100038

**Published:** 2023-12-06

**Authors:** Niels Møller Jensen, Tor Skibsted Clemmensen, Kamilla Pernille Bjerre, Omeed Neghabat, Lone Juul Hune Mogensen, Niels Ramsing Holm, Jouke Dijkstra, Evald Høj Christiansen, Steen Hvitfeldt Poulsen, Hans Eiskjær

**Affiliations:** a Department of Cardiology, Aarhus University Hospital, Aarhus, Denmark; b Department of Clinical Medicine, Aarhus University, Aarhus, Denmark; c Division of Image Processing, Department of Radiology, Leiden University Medical Center, Leiden, the Netherlands

**Keywords:** cardiac allograft vasculopathy, intravascular imaging, heart transplantation, optical coherence tomography, coronary artery

## Abstract

**Background:**

Cardiac allograft vasculopathy (CAV) is a prevailing complication following heart transplantation. We aimed to investigate if CAV causes equal vascular remodeling in the major coronary arteries using quantitative optical coherence tomography (OCT) and to explore the prognostic potential of OCT-derived measurements from each coronary artery.

**Methods:**

Sixty-four heart transplanted patients had a combined total of 114 full 3-vessel OCTs and coronary angiographies performed between 2013 and 2019. OCT pullbacks were categorized by angiographic CAV classification. Registration of disease progression was censored on July 1, 2022.

**Results:**

OCT recordings were classified as follows: no significant CAV, *n* = 73; mild CAV, *n* = 18; moderate CAV, *n* = 13; and severe CAV, *n* = 10. From intercoronary comparison of severe CAV, we found significant differences by both average lumen/intima ratio (*p* < 0.0001) and average intima/media ratio (*p* < 0.0001). The left descending artery (LAD) showed increasingly smaller luminal areas and larger intimal areas within CAV groups compared with the remaining coronary arteries. No differences were seen between major coronary arteries without significant CAV. LAD derived average intima/media ratio (hazard ratio (HR): 3.39; 95% confidence interval (CI): 1.33-8.63; *p* = 0.01) and average lumen/intima ratio (HR: 2.77; 95% CI: 1.09-7.05; *p* = 0.03) were the strongest predictors of CAV progression.LAD predictions were superior to predictions based on all 3 coronary arteries.

**Conclusions:**

LAD-derived OCT measurements were increasingly affected by CAV compared with the circumflex and right coronary artery. Average lumen/intima and intima/media ratios were the strongest predictors of CAV progression.

## Background

Heart transplantation (HTx) remains the optimal treatment for end-stage heart failure with a median survival exceeding 15-years post-transplantation.^1^ Cardiac allograft vasculopathy (CAV) is a highly prevalent coronary vascular complication affecting 47% of patients 10 years after HTx.^2^ The CAV pathogenesis is multifactorial and includes immunological processes causing characteristic diffuse intimal proliferation and lumen narrowing of the coronary vessels. Late stages of the disease compromise myocardial perfusion, ultimately leading to graft failure and death. Thus, CAV is the main cardiac cause of death beyond the first year after HTx.^3^

CAV is diagnosed and monitored by changes in coronary artery lumen using coronary angiography as the gold standard. However, coronary angiography is not sensitive to detect the early expansive intimal proliferation that does not initially reduce the lumen area.^4^, ^5^, ^6^ Optical coherence tomography (OCT) is an intravascular imaging modality that enables cross-sectional visualization of the coronary vessel wall compartments. The high spatial resolution allows for accurate estimation of lumen, intima, and media vessel proportions. The advantages of using OCT over angiography are well-documented in various settings^7^, ^8^ and previous studies have described the great prognostic value of OCT-determined CAV.^9^, ^10^, ^11^, ^12^ However, OCT is relatively costly and causes increased patient risks compared with coronary angiography. OCT examination requires more contrast therefore adding greater risks of allergic reactions and kidney damage. Furthermore, insertion of the guide wire introduces risks of dissection, perforation, and coronary spasms. Thus, maximizing the output from an OCT pullback and minimizing number of examinations are imperative. This study aims to quantify intercoronary vessel layer differences due to CAV. Additionally, we aim to investigate if procedural patient risks can be spared by only examining 1 vessel, or if multivessel examination provides incremental diagnostic and prognostic value.

## Methods

### Study population

In a retrospective cohort study, we included HTx patients from Aarhus University Hospital, Denmark, who underwent OCT examinations at their routine coronary angiography from September 6, 2013, until June 25, 2019. The cohort comprised patients included in previous studies.^10^, ^12^, ^13^ Inclusion criteria were >18 years of age, creatinine levels <200 μmol/liter, and successful OCT examinations of all 3 major coronary arteries. Patients provided informed written consent according to the principles of the Declaration of Helsinki. The Central Denmark Region Committees on Biomedical Research Ethics approved the study. The study was registered with ClinicalTrials.gov (NCT02077764 and NCT03583229). Data of clinical CAV progression were obtained from the Danish Electronic Health Records up until July 1, 2022.

In case of early CAV diagnosis, we considered switching immunosuppressive regime. In a substantial part of these patients, treatment with Everolimus had to be stopped. Mammalian target of rapamycin inhibitors have been shown to reduce early progression of intima proliferation, but there is not substantial evidence that introduction of Mammalian target of rapamycin in long-term patients with established CAV inhibits the progression of CAV at this stage.^14^ All patients at risk of developing CMV disease were treated with valganciclovir for at least 6 months as a routine.

### OCT image acquisition

Intravascular intervention was preceded by administration of intracoronary nitroglycerin (200-250 μg). After performing coronary angiography, intravenous heparin 5.000 IU was administered prior to OCT examination. OCT pullbacks were obtained from the left anterior descending artery (LAD), the left circumflex artery (LCX), and the right coronary artery (RCA) using Lunawave OFDI (Terumo, Tokyo, Japan), aiming for the longest possible pullbacks with coverage of up to 150 mm of each major branch. OCT recordings were performed during flushing with 15-20 ml contrast agent in each coronary artery with a pullback speed at 40 mm/sec. In case of inadequate image quality, the recordings were repeated after guiding catheter position adjustment.^10^, ^12^, ^13^

### OCT image analysis

**Figure 1:**
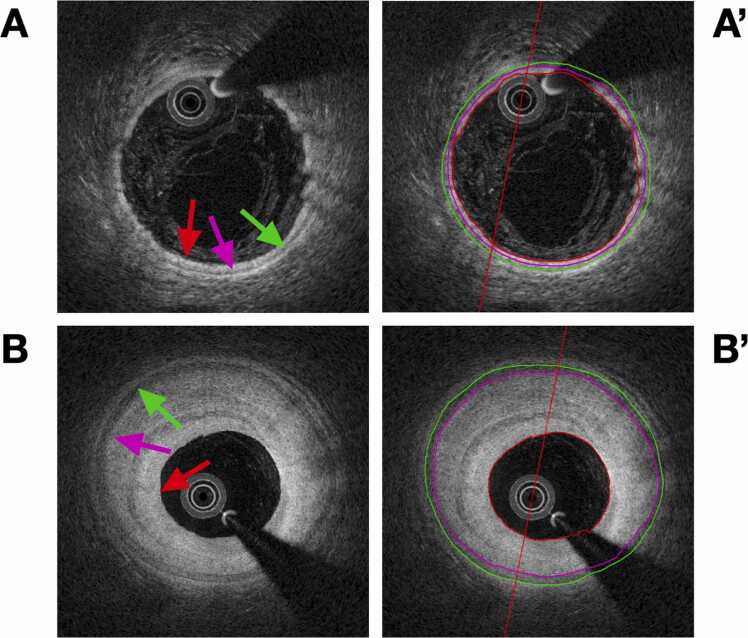
Quantitative optical coherence tomographic vessel layer analysis. (A) No significant cardiac allograft vasculopathy; (A′) vessel layer analysis; (B) severe cardiac allograft vasculopathy; (B′) vessel layer analysis. Red ring and arrow mark lumen-intima interface; pink ring and arrow mark intima-media interface; green ring and arrow mark media-adventitia interface.

All pullbacks were analyzed offline at 1-mm intervals using a customized version of the validated QCU-CMS software for OCT analysis (Medis Medical Imaging, the Netherlands). In each frame, we assessed lumen-intima interface, intima-media interface, and media-adventitia interface contour (Figure 1). From the contours, we extracted luminal area, intimal area, intimal thickness, and medial area. Lumen/intima ratios and intima/media ratios were calculated from the above measurements.

### Angiographic CAV assessment

Stratification of OCT measurements was based on CAV assessment by coronary angiography according to International Society for Heart and Lung Transplantation guidelines: CAV 0: not significant; CAV 1: mild, <50% left main stenosis and <70% stenosis of a major branch; CAV 2: moderate, ≤50% left main stenosis and ≥70% stenosis of a major branch; CAV 3: severe, ≥50% left main stenosis or ≥70% stenosis of 2 or more branches, or CAV 1 or CAV 2 with restrictive physiology of the left ventricle or reduced left ventricular ejection fraction ≤45%.^15^ Coronary angiography was performed in line with our center’s clinical guideline at 3 months, 1, 2, and 3 years after HTx. In case of normal examination, coronary angiography was performed every second year thereafter. In case of CAV development, coronary angiography was instead performed annually. CAV classifications were based on coronary angiograms at the first OCT examination and again at the point of each additional OCT examination.

### Outcomes

The outcome for the time-to-event analysis was clinical CAV progression corresponding to the clinical protocol at our center, defined as (1) registration of angiographic CAV progression according to International Society for Heart and Lung Transplantation criteria; (2) severe new coronary stenosis (>70%); (3) percutaneous intervention; (4) heart failure; and (5) myocardial infarction. Sudden deaths were censored and were not considered as an outcome. We included the first OCT examination from each patient to ensure longest available registration time. Regarding LAD vs 3-vessel prognostic comparison, a combined parameter was defined as the average of all 3 coronary vessel measurements.

### Statistical analysis

Histograms and Quantile-Quantile-plots were used to check for normality. Normally distributed data are presented as mean ± SD. Non-normally distributed data are presented as median and interquartile range. Categorical data are presented as counts and percentages. Between-group differences were assessed using Kruskal-Wallis test. We included multiple examinations per patient for the intercoronary comparison of vessel layer measurements. To accommodate for potential dependencies on patient level, a mixed model equation was applied with patient as random effect. Vessel layer measurements are presented for each CAV group and compared between coronary arteries. Distribution of residuals, random effects, and fixed effects were checked.

Time-to-event analyses were performed using Kaplan-Meier estimates and Cox proportional hazards method, hazard ratios (HR), and 95% CI. Only the first OCT examination per patient was included. Competing risk analyses were performed to ensure valid Kaplan-Meier curves. Adjusted Cox regression analysis was used to assess whether CAV at baseline had any effects on the result. Proportional hazards assumption in Cox regression models was assessed using log-log plots and plots of observed vs predicted. Optimal cut-off values were defined as the intersection points between sensitivity and specificity in receiver operating characteristics curves. We used intraclass correlation coefficients (ICC) and coefficient of variation (CV) to determine differences in vessel layer measurements comparing LCX and RCA, respectively, with the LAD.

Two-sided *p-*values *<*0.05 were considered statistically significant.

Analyses were performed using Stata/IC17 (StataCorp LP, College Station, TX) and GraphPad Prism 9 (GraphPad Software Inc., San Diego, CA).

## Results

### Clinical characteristics

At baseline, a total of 44 patients (69%) had CAV 0, 9 patients (14%) had CAV 1, 7 patients (11%) had CAV 2, and 4 patients (6%) had CAV 3. Table 1 presents patient demographics and pullback characteristics stratified according to angiographic CAV groups. Combined, baseline, and additional examinations counted 114 complete 3-vessel OCT examinations comprising 342 pullbacks with 17.729 analyzed frames, classified by the corresponding 114 coronary angiograms. We did not find any significantly higher prevalence of traditional risk factors, such as diabetes, hypertension, or hypercholesterolemia in CAV class 2-3 (Table 1).

**Table 1:** Optical Coherence Tomography Patient and Pullback Characteristics at Baseline

	CAV 0 *n* = 44	CAV 1 *n* = 9	CAV 2 *n* = 7	CAV 3 *n* = 4	Total *n* = 64
*Baseline patient characteristics*					
Men, %	73	89	71	50	73
Age, years	55 ± 11	53 ± 11	49 ± 6	64 ± 8	55 ± 11
Donor age, years	43 ± 12	50 ± 9	47 ± 11	36 ± 7	44 ± 12
Time since transplantation, years	5.0 [1.0-9.6]	9.0 [8.0-14.0]	8.7 [3.0-16.0]	19.0 [14.5-22.0]	7.0 [2.0-11.6]
Time until end of follow-up, years	5.9 [4.2-7.7]	7.7 [4.6-7.8]	2.9 [1.0-4.6]	4.6 [1.0-8.1]	5.6 [4.1-7.7]
Diabetes, %	14	44	0	0	16
Hypertension, %	84	78	57	100	81
DSA, %	24	25	14	50	25
Medication, %					
Prednisolone	45	33	29	0	39
Ciclosporine	20	11	43	100	27
Tacrolimus	80	89	57	0	73
Everolimus	23	22	29	0	22
Mycophenolate	88	67	71	25	80
Statins	86	67	100	75	84
ACE/AT II inhibitor	68	33	43	25	58
Furosemide or bumetanide	23	11	14	0	19
Aspirin	11	22	14	25	14
Biochemistry					
Creatinine, μmol/liter	100 [80-122]	104 [100-144]	109 [80-130]	129 [95-143]	103 [80-127]
Hemoglobin, mmol/liter	8.8 [7.9-9.3]	9.1 [8.1-9.3]	8.5 [7.7-10.2]	8.7 [8.3-9.4]	8.7 [7.9-9.4]
Total cholesterol, mmol/liter	4.8 [4.4-5.4]	4.6 [4.0-4.6]	3.9 [3.0-5.1]	5.0 [4.7-5.6]	4.7 [4.2-5.2]
Troponin T, ng/liter	11 [6-14]	11 [8-18]	6 [6-18]	16 [6-24]	11 [6-14]
NT-proBNP, ng/liter	217 [128-366]	322 [253-773]	423 [326-1441]	535 [358-1884]	308 [155-465]
CAV progression, %	25	11	71	50	30
Time until CAV progression, years	5.9 [4.2-7.7]	7.6 [4.6-7.8]	2.9 [1.0-4.6]	4.6 [1.0-8.1]	5.6 [4.1-7.8]
MACE after baseline, %					
Heart failure	7	22	0	0	8
Cardiovascular death	2	11	0	0	3
Rejection ≥ 2R	2	0	0	0	2
*OCT pullback characteristics*					
Total OCT pullbacks with corresponding angiographies, *n*					
LAD	73	18	13	10	114
LCX	73	18	13	10	114
RCA	73	18	13	10	114
Mean no. analyzed OCT frames, *n*					
LAD	53 ± 18	47 ± 15	43 ± 17	50 ± 16	50 ± 17
LCX	45 ± 14	39 ± 13	38 ± 16	41 ± 17	42 ± 14
RCA	65 ± 19	62 ± 19	51 ± 25	62 ± 23	63 ± 20

Abbreviations: ACE, angiotensin-converting enzyme; AT II = angiotensin II; CAV, cardiac allograft vasculopathy; DSA, donor-specific antibodies; LAD, left anterior descending artery; LCX, left circumflex artery; MACE, major cardiac event; NT-proBNP, N-terminal pro-B-type natriuretic peptide; OCT, optical coherence tomography; R, rejection; RCA, right coronary artery.

Values are %, mean ± SD or median [interquartile range].

We report 2 procedural complications related to all OCT examinations: one patient developed coronary spasms which resolved after administration of nitroglycerin. Another patient experienced ventricular fibrillation due to excessive use of contrast and was immediately successfully defibrillated. There were no coronary dissections, contrast-induced nephropathies, or allergic responses induced by OCT examinations.

### Quantitative comparison of OCT-derived vessel properties in angiographic CAV groups

**Figure 2:**
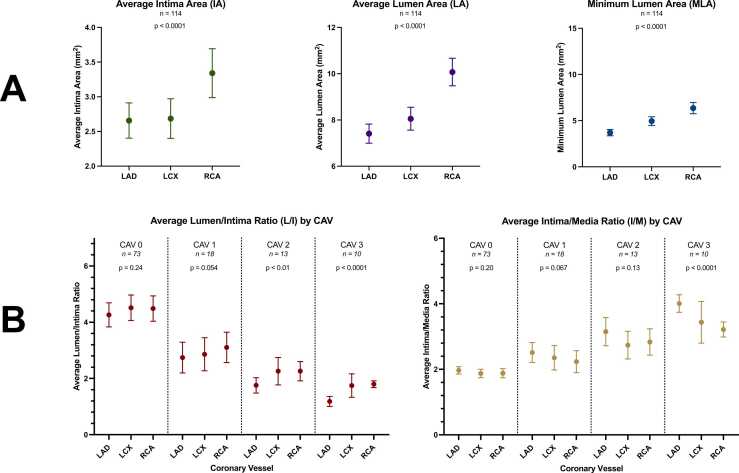
Between-vessel differences in vessel layers using optical coherence tomography. (A) Margin plots with mean and 95% confidence intervals (CI) demonstrating differences between vessels for average intima area (IA), average lumen area (LA), and minimum lumen area (MLA); (B) margin plots with mean and 95% CI demonstrating differences between vessels by cardiac allograft vasculopathy (CAV) for average lumen/intima ratio (L/I) and average intima/media ratio (I/M).

Table 2 displays mixed model comparisons of the absolute vessel measurements and ratios for LAD, LCX, and RCA within each angiographic CAV group. The results are graphically displayed in Figure 2.

**Table 2:** Quantitative OCT Analysis Stratified by Coronary Vessels and Angiographic CAV

	LAD *n* = 114	LCX *n* = 114	RCA *n* = 114	*p*-value^a^
Minimum lumen area, mm^2^				
CAV 0, *n* = 73^b^	3.93 (3.25-4.60)	5.30 (4.63-5.97)	6.83 (6.16-7.51)	<0.0001
CAV 1, *n* = 18^b^	3.80 (2.76-4.85)	4.95 (3.90-5.99)	4.35 (3.31-5.40)	0.17
CAV 2, *n* = 13^b^	3.74 (2.36-5.11)	4.47 (3.09-5.84)	7.36 (5.98-8.73)	<0.001
CAV 3, *n* = 10^b^	2.02 (0.42-3.63)	3.24 (1.63-4.84)	5.42 (3.82-7.03)	<0.001
Average lumen area, mm^2^				
CAV 0, *n* = 73^b^	7.91 (7.22-8.60)	8.34 (7.65-9.03)	10.24 (9.55-10.93)	<0.0001
CAV 1, *n* = 18^b^	6.50 (5.13-7.87)	7.85 (6.48-9.22)	8.82 (7.45-10.19)	<0.01
CAV 2, *n* = 13^b^	7.13 (5.56-8.70)	7.85 (6.28-9.42)	10.80 (9.23-12.36)	<0.0001
CAV 3, *n* = 10^b^	5.17 (3.59-6.75)	6.01 (4.43-7.59)	9.55 (7.97-11.13)	<0.0001
Average intima thickness, mm				
CAV 0, *n* = 73^b^	0.178 (0.156-0.199)	0.181 (0.160-0.203)	0.212 (0.190-0.233)	<0.001
CAV 1, *n* = 18^b^	0.251 (0.195-0.306)	0.275 (0.219-0.330)	0.270 (0.214-0.326)	0.45
CAV 2, *n* = 13^b^	0.365 (0.285-0.446)	0.317 (0.236-0.397)	0.356 (0.275-0.436)	0.17
CAV 3, *n* = 10^b^	0.462 (0.402-0.523)	0.374 (0.313-0.434)	0.426 (0.366-0.486)	<0.01
Average intima area, mm^2^				
CAV 0, *n* = 73^b^	1.95 (1.67-2.24)	2.01 (1.72-2.30)	2.63 (2.34-2.92)	<0.0001
CAV 1, *n* = 18^b^	2.56 (1.78-3.34)	3.14 (2.36-3.91)	3.20 (2.42-3.98)	0.071
CAV 2, *n* = 13^b^	4.00 (2.70-5.30)	3.63 (2.33-4.93)	4.74 (3.44-6.04)	0.063
CAV 3, *n* = 10^b^	4.51 (3.52-5.50)	3.86 (2.87-4.85)	5.25 (4.26-6.23)	<0.001
Average media area, mm^2^				
CAV 0, *n* = 73^b^	1.01 (0.89-1.12)	1.09 (0.97-1.21)	1.38 (1.26-1.50)	<0.0001
CAV 1, *n* = 18^b^	1.03 (0.79-1.26)	1.30 (1.06-1.53)	1.38 (1.15-1.62)	<0.01
CAV 2, *n* = 13^b^	1.34 (0.99-1.70)	1.39 (1.03-1.74)	1.79 (1.42-2.14)	<0.05
CAV 3, *n* = 10^b^	1.13 (0.91-1.36)	1.12 (0.90-1.35)	1.63 (1.41-1.86)	<0.0001
Ratios				
Average lumen/intima ratio				
CAV 0, *n* = 73^b^	4.55 (3.97-5.12)	4.80 (4.22-5.37)	4.77 (4.20-5.34)	0.24
CAV 1, *n* = 18^b^	2.87 (2.24-3.49)	2.99 (2.37-3.61)	3.23 (2.61-3.85)	0.054
CAV 2, *n* = 13^b^	1.91 (1.51-2.30)	2.41 (2.01-2.81)	2.41 (2.02-2.81)	<0.01
CAV 3, *n* = 10^b^	1.18 (0.96-1.41)	1.75 (1.52-1.97)	1.80 (1.57-2.02)	<0.0001
Average intima/media ratio				
CAV 0, *n* = 73^b^	1.95 (1.80-2.10)	1.86 (1.71-2.00)	1.86 (1.71-2.01)	0.20
CAV 1, *n* = 18^b^	2.50 (2.13-2.87)	2.34 (1.97-2.71)	2.22 (1.85-2.59)	0.067
CAV 2, *n* = 13^b^	3.05 (2.64-3.46)	2.64 (2.22-3.05)	2.73 (2.32-3.15)	0.13
CAV 3, *n* = 10^b^	4.01 (3.59-4.43)	3.45 (3.03-3.87)	3.22 (2.80-3.64)	<0.0001

Abbreviation: CAV, cardiac allograft vasculopathy.

Values are mixed model coefficients with 95% confidence intervals.

a *p*-value testing differences of mixed model coefficients between coronaries within each CAV-group.

b Number of OCT pullbacks for each individual coronary artery.

Minimum and average lumen area showed statistically significant differences between the 3 coronary arteries within all CAV groups except CAV 1. LAD values were lower compared with LCX whereas RCA values were substantially larger in all CAV groups.

Average intima thickness and average intima area showed statistically significant differences within CAV 0 and CAV 3 groups. Values increased in all 3 coronary vessels according to CAV grades. Average intima thickness values were higher for LAD compared with LCX and RCA, respectively, in CAV 2 and CAV 3 groups. Average media area values were different for CAV 0 and CAV 3. All values were higher for RCA. Average lumen/intima ratios decreased from CAV 0 through CAV 3 for all 3 coronary arteries. Average lumen/intima ratios for CAV 0 showed no significant difference between coronary arteries. The lumen/intima ratio was particularly reduced in LAD compared with LCX and RCA and especially with more advanced grades of CAV (mean reduction was approximately 20% larger for LAD compared with LCX and RCA from CAV 1 to CAV 3). Average intima/media ratios increased with CAV severity for all 3 coronary arteries. LAD ratios showed increasing differences compared to LCX and RCA with significant difference in CAV 3, similar to lumen/intima ratio assessment (Table 2).

### Prediction of CAV progression by quantitative OCT measurements

**Figure 3:**
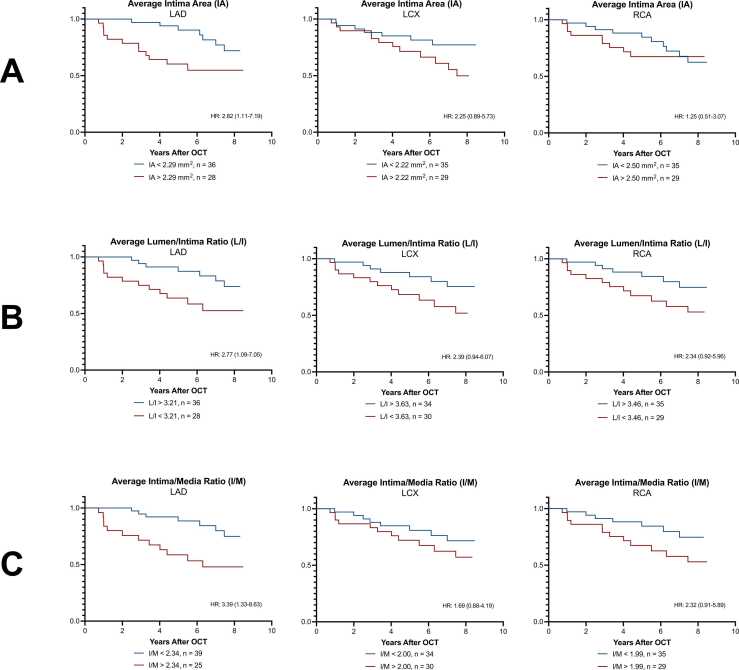
Prognostic value of OCT measurements from each coronary artery. Kaplan-Meier curves and hazard ratios (HR) for prediction of cardiac allograft vasculopathy (CAV) progression by optical coherence tomography (OCT) measurements derived from each coronary vessel. (A) Average intima area (IA); (B) average lumen/intima ratio (L/I); (C) average intima/media ratio (I/M).

The time-to-event analyses are presented in Table 3 and depicted in Figure 3. We included only the first OCT examination per patient, comprising a total of 64 3-vessel OCT examinations. Time between baseline and event registration for each CAV group was not significantly different (*p* = 0.16). During follow-up, 19 patients experienced CAV progression and 10 patients were censored because of death.

**Table 3:** Prediction of CAV Progression using OCT Measurements

	LAD *n* = 64	LCX *n* = 64	RCA *n* = 64	Average *n* = 64
Average lumen area				
Cutoff, mm^2^	6.81	8.14	9.25	8.24
Sensitivity	0.53	0.53	0.58	0.58
Specificity	0.56	0.53	0.58	0.56
Hazard ratio	1.59 (0.65-3.93)	0.97 (0.39-2.41)	1.68 (0.69-4.28)	1.24 (0.50-3.06)
*p*-value	0.312	0.946	0.243	0.636
Average intima thickness				
Cutoff, mm	0.23	0.20	0.21	0.22
Sensitivity	0.63	0.63	0.68	0.63
Specificity	0.67	0.60	0.62	0.67
Hazard ratio	2.65 (1.06-6.59)	2.39 (0.94-6.09)	2.07 (0.83-5.15)	2.74 (1.10-6.84)
*p*-value	0.036	0.068	0.118	0.031
Average intima area				
Cutoff, mm^2^	2.29	2.22	2.50	2.32
Sensitivity	0.63	0.63	0.53	0.63
Specificity	0.62	0.62	0.51	0.60
Hazard ratio	2.82 (1.11-7.19)	2.25 (0.89-5.73)	1.25 (0.51-3.07)	2.55 (1.00-6.50)
*p*-value	0.030	0.088	0.632	0.050
Average lumen/intima ratio				
Cutoff	3.21	3.63	3.46	3.76
Sensitivity	0.63	0.63	0.63	0.63
Specificity	0.62	0.62	0.62	0.62
Hazard ratio	2.77 (1.09-7.05)	2.39 (0.94-6.07)	2.34 (0.92-5.96)	2.66 (1.04-6.76)
*p*-value	0.033	0.068	0.074	0.040
Average intima/media ratio				
Cutoff	2.34	2.00	1.99	2.13
Sensitivity	0.68	0.58	0.63	0.63
Specificity	0.69	0.58	0.62	0.67
Hazard ratio	3.39 (1.33-8.63)	1.69 (0.68-4.19)	2.32 (0.91-5.89)	2.66 (1.05-6.77)
*p*-value	0.010	0.261	0.078	0.040

Hazard ratios presented with 95% confidence intervals.

Lumen area showed no significantly increased HR for single or combined vessel cut-off values. Intima thickness and intima area from LAD showed significant prediction of CAV progression similar to the combined three-vessel cut-off. Predictions using LCX and RCA intima measurements were nonsignificant. LAD-derived average lumen/intima and intima/media ratios showed predictions superior to intima measurements alone. Intima/media ratio for the LAD was ultimately the best-recorded predictor, even compared with the combined vessel cut-off.

### Differences from LAD

ICC and CV analysis showed stronger correlations for average lumen/intima ratios between LAD and LCX comparing with LAD and RCA. Average intima/media ratios from the LAD correlated well with both LCX and RCA (Table 4).

**Table 4:** Differences from LAD

	LCX			RCA		
	ICC (95% CI)	CV	Difference	ICC (95% CI)	CV	Difference
Minimum lumen area	0.41 (0.27-0.57)	59%	1.3 ± 2.4	0.12 (0.02-0.43)	75%	2.7 ± 3.5
Average lumen area	0.54 (0.41-0.67)	33%	0.6 ± 2.3	0.20 (0.07-0.43)	38%	2.7 ± 3.5
Average intima thickness	0.75 (0.66-0.82)	35%	−0.01 ± 0.08	0.69 (0.59-0.78)	37%	0.02 ± 0.1
Average intima area	0.72 (0.63-0.80)	43%	0.03 ± 1.1	0.60 (0.48-0.71)	48%	0.7 ± 1.5
Average lumen/intima ratio	0.77 (0.69-0.84)	37%	0.3 ± 1.3	0.80 (0.73-0.86)	38%	0.3 ± 1.2
Average intima/media ratio	0.75 (0.66-0.82)	25%	−0.2 ± 0.6	0.70 (0.60-0.78)	25%	−0.2 ± 0.6

Abbreviations: CI, confidence interval; CV, coefficients of variation; ICC, intraclass correlation coefficient.

Differences between OCT measurements including all 114 sets of pullbacks with LAD as reference.

## Discussion

This is the first study to evaluate intercoronary differences in CAV by comprehensive quantitative OCT evaluation. We report important findings regarding the nature of CAV distribution in HTx recipients:

1. LAD was relatively more affected by intimal proliferation and lumen narrowing compared with LCX and RCA in moderate to severe CAV groups.
2. Average lumen/intima and intima/media ratios were the strongest predictors of CAV progression.
3. The prognostic value of quantitative OCT for prediction of CAV progression was not increased by 3-vessel examination compared with 1-vessel examination using only LAD.

Most previous OCT studies of CAV have included only LAD pullbacks.^9^, ^16^, ^17^, ^18^, ^19^, ^20^ Few studies have included all 3 major coronary arteries without considering potential differences between coronary vessel properties.^21^ In recent studies from our center, we applied mixed model statistics to accommodate for potential dependencies when including multiple coronary arteries per patient.^12^, ^22^ From a clinical perspective, interventional cardiologists find themselves in an equivalent dilemma: is 1-vessel pullback sufficient for CAV evaluation, or does multivessel examination provide incremental value and therefore justify the additional procedural risks?

### Vessel properties

In the present study, we identified multiple differences in OCT measurements between the 3 major coronary arteries. Understandably, some can be explained by non–disease related differences of anatomy. For instance, RCA had relatively large luminal areas, particularly in vessels without significant CAV. The anatomical differences between coronary arteries can be approximated by comparing exclusively coronary arteries from the CAV 0 group. Not surprisingly, the major coronary arteries were significantly different in all absolute measurements within CAV 0. Intima measurements from CAV 1 and CAV 2 did however not differ between vessels, likely due to adverse intimal proliferation. Intimal proliferation affected LAD more severely in CAV 3 compared with LCX and RCA, as LAD had larger absolute thickness. Furthermore, the intercoronary differences in luminal area increased from CAV 0 to the CAV 3 group with a relatively more pronounced reduction of LAD and LCX measurements compared with RCA. For CAV assessment by OCT, it is important to consider the geometrical impact of using either thickness or area measurements with anatomically different vessels. Intimal area will increase with radius squared while thickness will only increase with radius. Consequentially, considering only intimal area or thickness individually could lead to incorrect CAV assessment due to anatomical differences.

Ratios provided a better understanding of CAV extent as they accommodate the differences between vessel proportions more precisely. In CAV 0, average lumen/intima and intima/media ratios showed no differences between the coronary arteries, implying the anatomical differences were relatively proportional. Most interestingly, average lumen/intima ratio for the LAD was progressively reduced compared to the other coronary arteries with CAV 1, 2, and 3. Similarly, average intima/media ratio revealed more extensive intimal proliferation relative to media area in LAD. Combined, these results suggest that the relative luminal reduction and intimal proliferation in CAV are more pronounced in LAD than LCX and RCA. Although further studies of the pathophysiology are required, we speculate if the intercoronary differences of CAV manifestations could be a consequence of flow differences. It is well-known that the left coronary system is more susceptible to atherosclerotic changes than the right coronary system.^23^, ^24^, ^25^ Chatzizisis et al similarly suggested that the increased susceptibility of the LAD to atherosclerotic changes could be explained by the hemodynamical differences between coronary arteries, as LAD is constituted by more regions with disturbed flow and thereby increased oscillatory shear stress.^26^

### Prediction of CAV progression by quantitative OCT measurements

**Figure 4:**
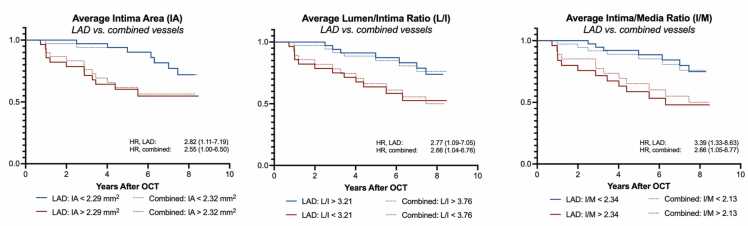
Prognostic value of OCT measurements: LAD vs combined vessels. Kaplan-Meier curves and hazard ratios (HR) for prediction of cardiac allograft vasculopathy (CAV) progression comparing LAD to the corresponding combined average coronary vessel measurements (combined). IA, intima area; L/I, lumen/intima ratio; I/M, intima/media ratio.

Our results suggest that LAD measurements are superior to corresponding measurements from LCX and RCA for prediction of CAV progression. Moreover, the prognostic value of isolated LAD measurements exceeded the combined 3-vessel average estimate in all measurements. Although application of individual cut-off values for each coronary artery was advantageous with 1-vessel predictions, it might have been less beneficial for the combined 3-vessel predictions. While absolute intima measurements and average lumen/intima ratios were significant predictors, average intima/media ratios provided numerically slightly better sensitivity, specificity, and HR for prediction of angiographic CAV progression (Figure 4).

A previous study found that OCT assessment of plaque morphology provided incremental value to angiographic assessment.^12^ Nevertheless, this study found quantitative OCT measurements (average intima area, average lumen/intima ratio, and average intima/media ratio) to be better prognostic markers compared with prediction by plaque morphology. In relation to our findings, we speculate if differences in plaque burden between vessels correlate to the quantitative differences regarding vessel layer morphology.

### Impact of results

Our results confirm the sufficiency of single-vessel LAD assessment by OCT for CAV evaluation after HTx. Additionally, we emphasize the importance of considering differences in anatomy between coronary arteries when interpreting OCT measurements.

In a clinical setting, there are advantages and disadvantages of 1-vessel focus compared with 3-vessel focus. Obviously, only evaluating LAD could lead to missing or underestimating vascular disease in LCX and the RCA. However, concerning CAV evaluation, we postulate that 3-vessel OCT examination does not add incremental value to neither prognostic nor rating of CAV severity. Therefore, it could be justified only introducing the OCT catheter into the LAD to spare procedural risks, time, and use of contrast agents.

Regarding application of specific cut-off values from our time-to-event analyses to prognostic OCT evaluation in a clinical setting, further and larger studies are warranted to test their validity and improve precision.

### Limitations

This study was based on a single-center experience. Only 4 patients with 10 full 3-vessel examinations had angiographic CAV 3. While multilevel statistics facilitated more robust results for the comparison of vessel properties, robustness of cut-off values from predictive analyses concerning severe CAV is modest.

Even though OCT examination of CAV status provides higher sensitivity, we applied the current golden standard with qualitative angiography assessment for CAV classification. It is likely that the premises of this methodology for CAV classification caused an underestimation of disease burden. Furthermore, angiographic event registration could have been less sensitive than OCT registration.

In this retrospective study design, we aimed to maximize robustness by including all available 3-vessel OCT examinations from our center regardless of time since HTx. In our relatively short follow-up, time since HTx was not a statistically significant predictor of CAV progression. Therefore, a prospective study with a longer follow-up should further characterize the actual progression rates in the coronary arteries over time.

### Conclusion

Assessed by quantitative vessel layer OCT analysis, average lumen/intima and intima/media ratios, in particular, revealed that LAD was increasingly more affected by CAV than LCX and RCA. LAD-derived lumen/intima and intima/media ratios were the strongest predictors of CAV progression. Average 3-vessel measurements did not exceed single-vessel LAD measurements in prediction of CAV progression.

## Disclosure statement

The authors did not receive funding and have no conflicts of interest to disclose in relation to the presented manuscript.
