# Supplementary material for: Cardiac allograft vasculopathy in heart transplanted recipients: The multivessel study
Source: JHLT Open. 2023 Dec 6;3:100038. doi: 10.1016/j.jhlto.2023.100038 (PMC11935483; doi:10.1016/j.jhlto.2023.100038)
Supplement: Supplementary file 1 — Supplementary material [file mmc1.docx]

# Twitter Handle

1st author and corresponding author: @nielsmollerjens

Department of Cardiology Research Aarhus University Hospital: @AUHCardio

# Tweet (at least 210 characters)

HTx: By means of OCT, Danish researchers investigated differences of CAV manifestation in the major coronary arteries. LAD is more affected by CAV compared with LCX and RCA and offers superior prognostication of CAV progression🫀

*228 characters
